# Characterisation of the Plasma and Faecal Metabolomes in Participants with Functional Gastrointestinal Disorders

**DOI:** 10.3390/ijms252413465

**Published:** 2024-12-16

**Authors:** Karl Fraser, Shanalee C. James, Wayne Young, Richard B. Gearry, Phoebe E. Heenan, Jacqueline I. Keenan, Nicholas J. Talley, Warren C. McNabb, Nicole C. Roy

**Affiliations:** 1 AgResearch, Tennent Drive, Palmerston North 4442, New Zealand; 2 The Riddet Institute, Massey University, Palmerston North 4474, New Zealand; 3 High-Value Nutrition National Science Challenge, Auckland 1023, New Zealand; 4 School of Food and Advanced Technology, Massey University, Palmerston North 4472, New Zealand; 5 Department of Medicine, University of Otago, Christchurch 8011, New Zealand; 6 Department of Surgery, University of Otago, Christchurch 8011, New Zealand; 7 School of Medicine and Public Health, The University of Newcastle, Callaghan, Newcastle 2308, Australia; 8 Department of Human Nutrition, University of Otago, Dunedin 9016, New Zealand

**Keywords:** metabolomics, gut-brain, constipation, biomarkers, diarrhoea, LC-MS

## Abstract

There is evidence of perturbed microbial and host processes in the gastrointestinal tract of individuals with functional gastrointestinal disorders (FGID) compared to healthy controls. The faecal metabolome provides insight into the metabolic processes localised to the intestinal tract, while the plasma metabolome highlights the overall perturbances of host and/or microbial responses. This study profiled the faecal (*n* = 221) and plasma (*n* = 206) metabolomes of individuals with functional constipation (FC), constipation-predominant irritable bowel syndrome (IBS-C), functional diarrhoea (FD), diarrhoea-predominant IBS (IBS-D) and healthy controls (identified using the Rome Criteria IV) using multimodal LC-MS technologies. Discriminant analysis separated patients with the ‘all constipation’ group (FC and IBS-C) from the healthy control group and ‘all diarrhoea’ group (FD and IBS-D) from the healthy control group in both sample types. In plasma, almost all multimodal metabolite analyses separated the ‘all constipation’ or ‘all diarrhoea’ group from the healthy controls, and the IBS-C or IBS-D group from the healthy control group. Plasma phospholipids and metabolites linked to several amino acid and nucleoside pathways differed (*p* < 0.05) between healthy controls and IBS-C. In contrast, metabolites involved in bile acid and amino acid metabolism were the key differentiating classes in the plasma of subjects with IBS-D from healthy controls. Faecal lipids, particularly ceramides, diglycerides, and triglycerides, varied (*p* < 0.05) between healthy controls and the ‘all constipation’ group and between healthy controls and ‘all diarrhoea’ group. The faecal and plasma metabolomes showed perturbations between constipation, diarrhoea and healthy control groups that may reflect processes and mechanisms linked to FGIDs.

## 1. Introduction

Functional gastrointestinal disorders (FGID), also known as disorders of gut-brain interaction [1], including irritable bowel syndrome (IBS) [2], are common and affect 7–21% of people worldwide, impacting the quality of life and burdening health care systems [3]. Dietary intolerance, visceral hypersensitivity, immune activation, dysmotility, gut microbial dysfunction, impaired mucosal structure of the intestine, and a dysregulated gut-brain axis are important for understanding the processes and mechanisms that result in FGIDs [4]. The gut microbiota plays a critical role in regulating gastrointestinal physiology, resistance to pathogen colonisation, intestinal development and the production of physiologically important metabolites from the diet. Thus, recent evidence indicates its important role in FGID [5,6]. A number of studies have shown clear differences in the gut microbial composition between individuals with FGID and healthy individuals [6,7].

Metabolites provide a readout reflective of host-microbial metabolic processes [8,9,10]. For this reason, studies rely on characterising the concentration or relative intensity of metabolites and proteins in faecal samples to gather insights about host metabolism and to provide insight into interactions between dietary-derived components and the microbiome in the gastrointestinal tract. Similarly, the plasma metabolome provides insights into circulating metabolites that are transported from the gastrointestinal lumen to the rest of the body. It can provide insight into the underlying human and/or microbial physiological responses [11].

Faecal metabolites linked to fatty acid, amino acid, bile acid, and fructose metabolism have been shown to differ in relative intensities between individuals with FGID and healthy controls [6,12]. These metabolites can exert major effects throughout the body. For example, amino acids, as precursors to numerous important metabolic functions, such as tryptophan, the precursor to serotonin, which could be linked to interactions of the gut-brain axis, intestinal immune response, and T cell proliferation [13], have important links to IBS symptoms. The faecal microbiome can also influence the metabolite profiles observed, and IBS has been shown to influence microbial community composition, function, and metabolite profiles, with changes between different IBS phenotypes [14]. The plasma metabolome has seldom been characterised in individuals with FGID compared to the faecal metabolome. However, in healthy individuals, up to 46% of the variation in the plasma metabolome in humans may be explained by changes in the gut microbiota [15].

This primary aim was to measure relative intensity differences in plasma and faecal samples using multiple LC-MS metabolomic methods (multimodal; i.e., metabolites were chromatographically resolved using three different chromatographic modes on two separate extracts). Anticipated outcomes were to better understand the metabolic pathways and processes that differ between individuals with either ‘all constipation’ (FC + IBS-C) group, ‘all diarrhoea’ (FD + IBS-D) group, or IBS subgroups, compared to healthy controls.

## 2. Results

Symptom questionnaires based on the Rome Criteria IV clustered participants providing faecal samples as ‘all constipation’ (FC + IBS-C, *n* = 58), ‘all diarrhoea’ (FD + IBS-D, *n* = 66) and healthy control (*n* = 97) groups. For the collected plasma samples, participants were again clustered as ‘all constipation’ (FC + IBS-C, *n* = 53), ‘all diarrhoea’ (FD + IBS-D, *n* = 60) and healthy control (*n* = 93) groups.

A total of 221 faecal and 206 plasma samples were analysed. Non-polar metabolite analysis identified 428 annotated lipids belonging to 16 different lipid classes in plasma samples (Supplementary Table S1) and 421 annotated lipids belonging to 23 different lipid classes in faecal samples (Supplementary Table S2). Semi-polar metabolite analysis of the plasma samples detected 1004 features, with 125 metabolites annotated (Supplementary Table S3), while faecal samples detected 1203 features, with 140 metabolites annotated (Supplementary Table S4). Polar metabolite analysis of the plasma samples detected 876 features, with 94 metabolites annotated (Supplementary Table S5), while in faecal samples 694 features were detected, with 78 metabolites annotated (Supplementary Table S6).

Multivariate analysis (PCA and PLS-DA) was performed on these groups and on the IBS-C and IBS-D phenotypes vs. the healthy controls to investigate further IBS metabolite-related mechanisms for both plasma and faecal samples. The PCA plots revealed no obvious clustering of constipation, diarrhoea, or IBS-alone groupings in multimodal plasma or faecal sample analyses. Thus, supervised multivariate modelling using PLS-DA was performed to create and refine (data-driven) multivariate models and determine metabolites that may contribute to differences between groups.

These data-driven supervised approaches can distinguish if metabolites have different relative intensities between the groups, which individually may not reach the *p* < 0.05 significance threshold. However, changes in the relative intensities of many metabolites, when combined, can reveal underlying mechanisms where multiple metabolites are altered within the same or similar metabolic pathways. Significant (*p* < 0.05) multivariate models were only generated for the faecal and plasma samples for the ‘all constipation’ or ‘all diarrhoea’ groups compared to the healthy control group. These analyses also revealed that for plasma metabolites (but not faecal metabolites), the individual subgroups of healthy controls (*n* = 97) vs. IBS-C (*n* = 23) or vs. IBS-D (*n* = 48) generated significant (*p* < 0.05) multivariate models. Further analyses of the plasma and faecal metabolomes of the subgroups were carried out with the ‘all constipation’ or ‘all diarrhoea’ group compared to the healthy control groups, except for the plasma metabolome, where the IBS subgroups were compared to the healthy control group. The results are presented in more detail below.

### 2.1. Comparison Between Constipation and Healthy Control Groups

#### 2.1.1. Plasma and Faecal Non-Polar Metabolites

**Figure 1:**
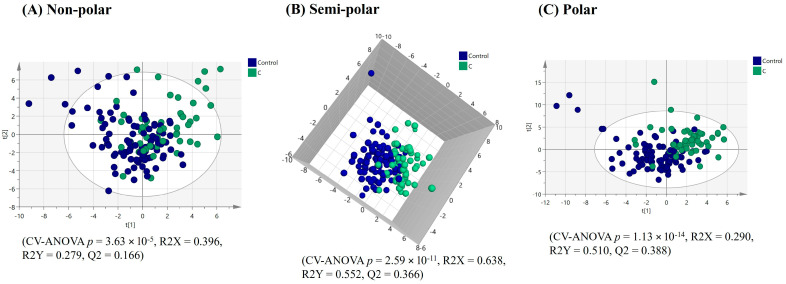
PLS-DA score plots of plasma multimodal metabolomics ((**A**) non-polar, (**B**) semi-polar and (**C**) polar metabolite) analyses for ‘all constipation’ compared to the healthy control group.

PLS-DA model refinement yielded a significant model based on the top 51 VIP score plasma lipids (VIP > 1.36) from the original model, demonstrating the separation of the ‘all constipation’ individuals (FC + IBS-C) from the healthy controls. However, the model was characterised by a weak Q2 value (Figure 1A). Univariate statistics on the complete plasma lipid profile revealed five lipids with significantly differing relative intensities and having a fold-change > ±1.3 between the healthy controls and the ‘all constipation’ group. Of these all four significant triacylglycerides were lower in relative intensities in the ‘all constipation’ group, while one phosphoethanolamine was higher in relative intensity in the ‘all constipation’ group (Table 1).

**Figure 2:**
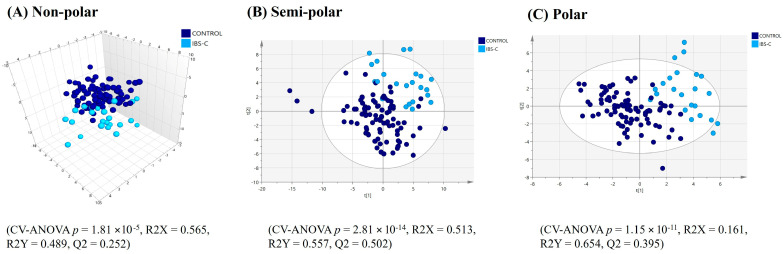
PLS-DA score plots of plasma multimodal metabolomics ((**A**) non-polar, (**B**) semi-polar and (**C**) polar metabolite) analyses for IBS constipation compared to the healthy control group.

A further model comparing only the IBS-C individuals and healthy controls yielded an improved and significant model based on the top 56 VIP score plasma lipids (VIP > 1.25) with a stronger Q2 than the ‘all constipation’ modelling (Figure 2A). Univariate statistics on the complete plasma lipid profile revealed 25 significant lipids with different relative intensities and a fold-change > ±1.3, between the healthy controls and the IBS-C group. Notably, of these, all six significant triacylglycerides had lower relative intensities, whereas 19 phospholipids had higher relative intensities in the IBS-C group (Table 2).

**Figure 3:**
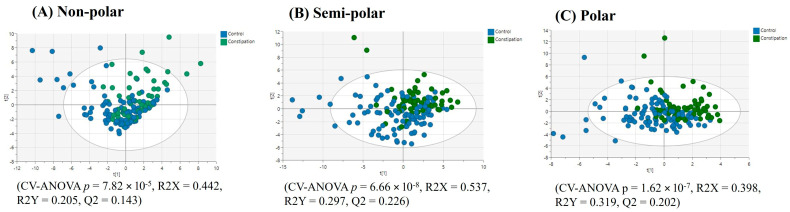
PLS-DA score plots of faecal multimodal metabolomics ((**A**) non-polar, (**B**) semi-polar and (**C**) polar metabolite) analyses for ‘all constipation’ compared to the healthy control group.

For the faecal samples, a PLS-DA model based on the top 42 VIP score faecal lipids (VIP > 1.42) showed the lipid profiles of the individuals in the ‘all constipation’ group were significantly distinct from that of the healthy controls, albeit with an overlap of the confidence intervals. However, the model was characterised by a weak Q2 value (Figure 3A). Univariate statistics on the complete faecal lipid profile revealed 24 lipids with significantly differing relative intensities and having a fold-change > ±1.3 between the healthy controls and the ‘all constipation’ group. Notably, of these, six significant diacylglycerides were lower in relative intensities in the ‘all constipation’ group, while nine ceramides were higher in relative intensities in the ‘all constipation’ group (Table 3).

#### 2.1.2. Plasma and Faecal Semi-Polar and Polar Metabolites

The PLS-DA analysis (after model refinement) based on the top 111 VIP score plasma semi-polar metabolites (VIP > 1.59) from the original model demonstrated significant separation of the ‘all constipation’ and healthy control groups (Figure 1B). A further PLS-DA-refined model comparing the IBS-C group and the healthy control group based on the top 101 VIP score plasma semi-polar metabolites (VIP > 1.60) from the original model yielded improved and significant visual separation (Figure 2B).

The PLS-DA analysis (after model refinement) based on the top 102 VIP score plasma polar metabolites (VIP > 1.53) showed separation between the ‘all constipation’ and healthy control groups (Figure 1C). A further PLS-DA refined model comparing IBS-C individuals with healthy controls based on the top 80 VIP score plasma polar metabolites (VIP > 1.54) yielded an improved, significant visual separation (Figure 2C).

**Figure 4:**
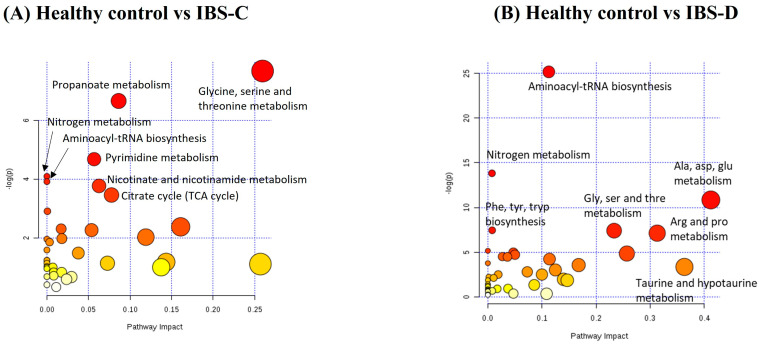
Metabolite set enrichment analysis for key differential polar and semi-polar metabolites measured in plasma for (**A**) IBS constipation compared to the healthy control group and (**B**) IBS diarrhoea compared to the healthy control group.

Given the significant models obtained for the IBS-C group, metabolite set enrichment analysis (MSEA) was conducted to elucidate differential pathways between the IBS-C group and healthy controls using 39 annotated metabolites selected from the PLS-DA models (refer to Figure 2B,C). A list of metabolites is presented in Supplementary Table S7. MSEA (Figure 4A) revealed that seven pathways were significantly impacted (*p* < 0.05): amino acid (glycine, serine and threonine) metabolism; propanoate metabolism; pyrimidine metabolism; nitrogen metabolism; amino-tRNA biosynthesis; nicotinate and nicotinamide metabolism; and the citric acid cycle. The most impacted, significant pathways were glycine, serine and threonine metabolism. To further understand the connections between these metabolic pathways, metabolite networks were constructed using the Cytoscape Metscape tool (Supplementary Figure S1). This analysis revealed that glycine was a hub linking to bile acid metabolism, nicotinamide metabolism, and other amino acid pathways such as tryptophan metabolism in participants with IBS-C.

The PLS-DA analysis (after model refinement) based on the top 55 VIP score faecal semi-polar metabolites (VIP > 1.67) from the original model demonstrated significant separation of the ‘all constipation’ group from the healthy control group, albeit with an overlap in PLS-DA groups (Figure 3B). MSEA was not performed for the faecal metabolome as its composition reflects both the host and/or microbial metabolism and thus compromises the single organism metabolism approach of MSEA. Univariate analysis was conducted to understand the relative intensity differences of faecal metabolites in subjects with constipation or diarrhoea. Seven metabolites significantly differed in relative intensity with a fold change > ±1.3 between the ‘all constipation’ group and healthy controls (Table 4). These significant faecal metabolites, notably choline and glycocholic acid, had lower relative intensity in the ‘all constipation’ group compared to the healthy control group. Only pimelic acid had a higher relative intensity in the participants in the ‘all constipation’ group compared to healthy controls.

Similar to the lipid and semi-polar metabolite results for faecal samples, PLS-DA analysis based on the top 37 VIP faecal polar metabolites (VIP > 1.66) showed significant separation between ‘all constipation’ and healthy control groups, albeit with an overlap in confidence intervals (Figure 3C). The univariate analysis only detected four known metabolites with significantly differing relative intensities and a fold-change of >±1.3 between ‘all constipation’ and healthy control groups, and all were higher in the ‘all constipated’ group (Table 5).

### 2.2. Comparison Between Diarrhoea and Healthy Control Groups

#### 2.2.1. Plasma and Faecal Non-Polar Metabolites

**Figure 5:**
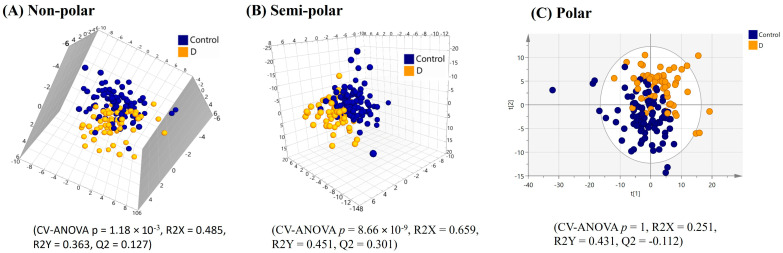
PLS-DA score plots of plasma multimodal metabolomics ((**A**) non-polar, (**B**) semi-polar and (**C**) polar metabolite) analyses for ‘all diarrhoea’ compared to the healthy control group.

**Figure 6:**
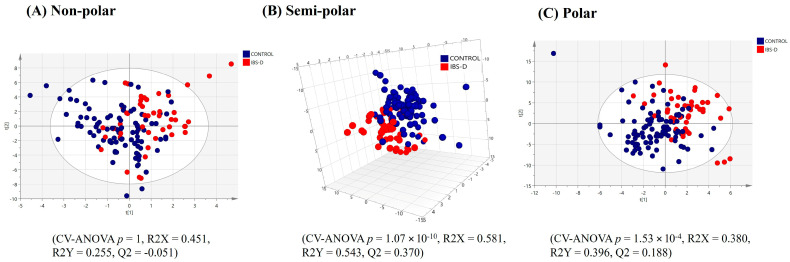
PLS-DA score plots of plasma multimodal metabolomics ((**A**) non-polar, (**B**) semi-polar and (**C**) polar metabolite) analyses for IBS diarrhoea compared to the healthy control group.

A PLS-DA model (after model refinement) based on the top 53 VIP score plasma lipids (VIP > 1.41) showed visual separation of the ‘all diarrhoea’ (FD +IBS-D) group compared to the healthy controls (Figure 5A), but a low Q2 value indicating a weak model. Univariate statistics on the complete plasma lipid profile revealed no lipids with significantly differing relative intensities and having a fold-change > ±1.3 between the ‘all diarrhoea’ group and healthy controls. A model comparing the relative intensity of plasma lipids from the IBS-D group and healthy controls shows a poor, non-significant visual separation (Figure 6A), and again, univariate analysis showed no significant differences for this comparison.

**Figure 7:**
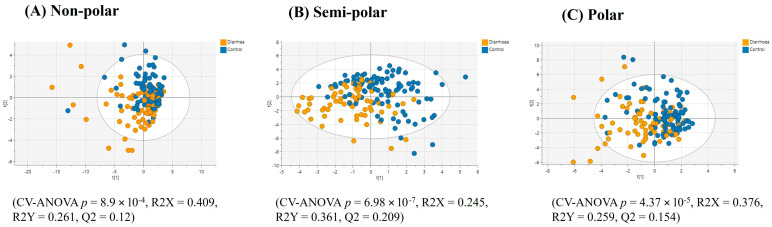
PLS-DA score plots of faecal multimodal metabolomics ((**A**) non-polar, (**B**) semi-polar and (**C**) polar metabolite) analyses for ‘all diarrhoea’ group compared to the healthy control group.

The PLS-DA model based on the top 44 VIP score faecal lipids (VIP > 1.43) showed that the lipid profile of the ‘all diarrhoea’ group was significantly distinct from that of the healthy controls, albeit with an overlap of the confidence intervals (Figure 7A). The univariate analysis of the complete faecal lipid profile revealed 19 lipids with significantly differing relative intensities (fold-change > ±1.3) between the ‘all diarrhoea’ group and the healthy controls. Of these significant lipids, 10 ceramides were higher in relative intensities in the ‘all diarrhoea’ group, while only (*O*-Acyl)-ω-hydroxy fatty acid was lower in relative intensity in the ‘all diarrhoea’ group (Table 6).

#### 2.2.2. Plasma and Faecal Semi-Polar and Polar Metabolites

The PLS-DA analysis based on the top 110 VIP score semi-polar metabolites (after model refinement, VIP > 1.60) in plasma samples showed significant separation between the ‘all diarrhoea’ and healthy control groups (Figure 5B). A further refined model based on the top 106 VIP score semi-polar metabolites (VIP > 1.60), comparing the healthy controls to the IBS-D group, yielded an improved, significant visual separation (Figure 6B).

The PLS-DA analysis (after model refinement) based on the top 98 VIP score plasma polar metabolites (VIP > 1.41) showed a poor, non-significant separation between the ‘all diarrhoea’ and healthy control groups (Figure 5C). A further PLS-DA refined model comparing the IBS-D group against the healthy control group based on the top 110 VIP score plasma polar metabolites (VIP > 1.39) yielded a weak significant visual separation (Figure 6C).

As observed in the IBS-C group, significant models were generated with plasma metabolite data, and the IBS-D group was separated from the healthy control group. Combining the polar and semi-polar metabolite lists for the IBS-D vs. healthy control comparison for MSEA with 42 annotated metabolites revealed that 17 pathways were significantly impacted (*p* < 0.05, Figure 4B and Table 7). Of note, glycine, serine, and threonine metabolism, along with many other amino acid pathways, were also important, as were the taurine and hypotaurine pathways, which are linked to bile acid production. The list of metabolites used for the MSEA is documented in Supplementary Table S8.

To further understand the connections between these metabolic pathways, metabolite networks were constructed using the Cytoscape Metscape tool (Supplementary Figure S2). This analysis revealed that the key regulatory hubs were mostly related to protein and amino acid metabolism.

For faecal samples, the PLS-DA analysis based on the top 54 VIP semi-polar metabolites (VIP > 1.62) showed significant separation between ‘all diarrhoea’ and healthy control groups, again albeit with an overlap in PLS-DA models (Figure 7B). Univariate analysis revealed eight metabolites with significantly different relative intensities (fold change > ±1.3) between the ‘all diarrhoea’ group and healthy controls (Table 8). Of these significant faecal metabolites, everninic acid (as observed in the ‘all constipation’ group) had lower relative intensity in the ‘all diarrhoea’ group compared to the healthy control group. Five of the eight metabolites had a higher relative intensity in the participants in the ‘all diarrhoea’ group compared to healthy controls.

Similar to the faecal lipid and semi-polar results, the PLS-DA analysis based on the top 33 VIP faecal polar metabolites (VIP > 1.60) showed significant separation between ‘all diarrhoea’ and healthy control groups (Figure 7C). Univariate analysis revealed 10 metabolites with significantly different relative intensities (fold change > ±1.3) between the ‘all diarrhoea’ group and healthy controls (Table 9). Of these significant faecal metabolites, all 10 had lower relative intensities in the ‘all diarrhoea’ group compared to the control group.

## 3. Discussion

Here, a multimodal (HILIC for polar metabolites, reverse-phase for semi-polar metabolites, and lipidomics for non-polar metabolites) metabolomic approach of faecal and plasma samples showed differences in metabolite relative intensities between individuals with either constipation (FC + IBS-C) or diarrhoea (FD + IBS-D) group compared to the healthy control group. Faecal lipid metabolism was the most important metabolites to the ‘all constipation’ and ‘all diarrhoea’ groups in differentiating from the healthy controls. Minor differences in semi-polar and polar metabolites were also noted in these comparisons.

Furthermore, in plasma, separation of the IBS groups from the healthy control group was observed using multivariate and univariate analyses. Plasma phospholipids and amino acid-related metabolites were key in differentiating IBS-C individuals from healthy controls. Meanwhile, plasma amino acid and bile acid metabolites were important for differentiating IBS-D individuals from healthy controls.

### 3.1. Lipid Profiles in Individuals with Disorders of Gut-Brain Interactions

This study showed that only five plasma lipids differed in relative intensity for the ‘all constipation’ comparison and none for the ‘all diarrhoea’ comparison. However, 25 plasma lipids had differing relative intensities between the groups when comparing IBS-C and healthy controls. Of these, all lipids had higher relative intensities in the IBS-C individuals, and 19 out of 25 were phospholipids, while six lipids (all triacylglycerides) had lower relative intensities in the IBS-C group. This was not the case for the plasma lipid comparison between IBS-D vs. healthy control groups, highlighting that plasma lipids were impacted in IBS-C condition rather than in diarrhoea (‘all diarrhoea’ or IBS-D). These findings agree with the increased concentrations of ceramides, glycosphingolipids, diglycerides, and triglycerides reported in mucosal biopsies and plasma samples of individuals with IBS compared to healthy controls [16] and also in individuals with myalgic encephalomyelitis/chronic fatigue syndrome coupled with IBS [17].

The univariate analysis revealed that 24 faecal lipid species differed In relative intensities for the ‘all constipation’ group, with ceramides and phospholipids higher in the ‘all constipation’ samples, while glycerolipids (di- and triacylglycerides) were lower in the ‘all constipation’ group. For the ‘all diarrhoea’ vs. healthy control comparison, 19 lipids differed in relative intensities, the predominant lipid species including ceramides and glycerolipids, with 18 of the discriminating lipids species higher in the ‘all diarrhoea’ group than the healthy controls and only one OAHFA lower in the ‘all diarrhoea’ group. Thus, faecal lipids were important discriminants of the ‘all constipation’ or ‘all diarrhoea’ groups compared to the healthy control group, particularly ceramides, triglycerides, and diglycerides. Other studies have also associated specific annotated metabolites with intestinal ailments [6,7]. Jeffery et al. showed that faecal glycerophospholipids and oligopeptides were important for differentiating IBS and healthy participants [6].

In the current study, triglycerides were more abundant in the ‘all diarrhoea’ group. This finding agrees with existing literature, where elevated faecal triglycerides have been linked to bile acid malabsorption and diarrhoea conditions via the FXR receptor [18]. The FXR receptor is crucial for producing primary bile acids and regulates lipid and glucose homeostasis via various mechanisms, including increasing triglyceride hydrolysis [19,20]. FXR-deficient mice showed hypertriglyceridemia and impaired bile acid homeostasis [20,21], and individuals with hypertriglyceridemia have shown disruptions in ileal bile acid reabsorption [20,22]. Thus, disruptions to the FXR receptor or bile acid hepatic circulation commonly associated with diarrhoea-predominant conditions could be linked to increased triglyceride concentrations in faecal samples. Concurrent with the increased relative intensity of triglycerides reported here, increased faecal bile acid concentrations were observed in participants in the ‘all diarrhoea’ group compared to participants in the ‘all constipation’ or healthy control groups of the same cohort [23]. These findings support a positive association between bil” aci’ and triglyceride metabolism in FGID.

Nine faecal ceramides were more abundant in the ‘all constipation’ group than the healthy control group, with all of these ceramides containing medium-length fatty acid sidechains (carbon chain lengths ~C12–C18), with five of the nine ceramides containing odd-chain fatty acids, which are commonly microbially-produced fatty acids. Four of the 11 ceramides with different relative intensities contained fatty acids longer than C18 in the ‘all diarrhoea’ group compared to healthy controls. Ceramides are waxy lipids associated with pain sensitivity, cell toxicity, inflammation, and various diseases, such as metabolic disorders, Alzheimer’s disease, insulin resistance, and inflammatory bowel diseases [16,17]. Kajander et al. showed that the relative intensity of lipids in the ceramide and sphingomyelin pathways was increased in IBS participants (all subtypes combined) compared to healthy controls [16]. In individuals with myalgic encephalomyelitis or chronic fatigue syndrome (often associated with IBS), changes in the gut microbiome were associated with increased faecal lipopolysaccharide concentrations [16]. This increase may trigger sphingomyelinases that, when hydrolysed, form ceramides [17,24], contributing to oxidative stress and intestinal barrier dysfunction [16]. Furthermore, the lipotoxicity of ceramides can degrade other key lipid structures, triggering IBS symptoms [16]. Additionally, evidence suggests that bacterial pathogens can manipulate the structural and signalling properties of ceramides to promote pathogenic bacterial colonisation [25], highlighting a further possible link to FGIDs.

However, not all types or concentrations of ceramides are toxic. Sphingolipids, the wider group encompassing ceramides, are important in cell membrane structure and signalling [26]. For example, ceramides have been suggested to induce cell apoptosis in response to stressors such as radiation or chemotherapy, acting as ligands that bind to and regulate enzyme activity and many other intracellular functions [26]. Therefore, ceramides may increase lipotoxicity only during other stressors, such as those commonly associated with FGID.

Odd sidechain ceramides were present at higher relative intensity in the ‘all constipation’ group compared to even sidechain ceramides in the ‘all diarrhoea’ group. Published studies have shown that odd sidechain fatty acids are not a human metabolic product and are obtained from the diet or through microbial modification [27]. The differences in sidechains and evidence supporting the importance of ceramides in other diseases [28,29] warrant further research. The lack of literature regarding the role of lipids in FGID and their importance to intestinal tissue integrity and metabolism, together with the findings here, suggests that the importance of lipids has been overlooked, and further quantitative analysis is warranted.

### 3.2. Semi-Polar and Polar Metabolite Profiles in Individuals with Disorders of Gut-Brain Disorders

Analysis of plasma metabolites revealed significant PLS-DA models for the ‘all constipation’ group or IBS-C group compared to the healthy control group. MSEA highlighted perturbations in amino acid and pyrimidine, vitamin and citric acid cycle pathway metabolites. Purines are a type of pyrimidine structure, and a longitudinal study found changes in purine metabolism in IBS-C and IBS-D individuals compared to healthy controls [30]. Pathway analysis pointed to plasma glycine as a potential regulating metabolite for perturbation in metabolism seen in IBS, linking with vitamin (nicotinamide) metabolism and bile acid metabolism [23]. Some studies have shown that IBS individuals have lower levels of some vitamins compared to non-IBS individuals [31]. Dietary glycine supplementation in both mice and pigs enhances intestinal barrier function, reduces inflammation and alters intestinal microbial composition [32,33].

From the diarrhoea perspective, some complex differences in plasma semi-polar metabolite profile were observed between the ‘all diarrhoea’ compared to the healthy control group. In the IBS-D subgroup, 17 differential pathways were impacted compared to those in the healthy control group. Again, amino acid metabolism and bile acid-related pathways were observed to be important and interconnected.

Amino acid metabolism plays a crucial role in the pathophysiology of inflammatory bowel-related conditions such as IBD. Some amino acids can modulate inflammation by regulating macrophages and inhibiting many inflammatory pathways in macrophages, such as NF-κB, STAT1, and STAT5 [34]. They (and their metabolites) also play an important role in the growth and function of T cells through the intracellular uptake of various amino acid transport proteins widely expressed on the T cell membrane, which are responsible for maintaining T cell survival and proliferation. In addition, amino acid post-translational modifications, such as methylation and acetylation, are important players in directly regulating T cell typing at the genetic level [34].

Little has been published on amino acid metabolism and IBS specifically. However, alterations in amino acid metabolism have been observed, particularly involving glutamine, arginine and tryptophan, in inflammatory bowel conditions. Glutamine, a conditionally essential amino acid, is a major energy source for intestinal epithelial cells and plays a role in maintaining intestinal barrier function [35]. Reduced levels of glutamine have been associated with increased intestinal permeability and inflammation in IBS, and glutamine supplementation as part of a low FODMAP diet is promising for enhancing the amelioration of symptoms achieved with the FODMAP diet in IBS individuals [36]. Arginine, another important amino acid, contributes to nitric oxide production and modulation of immune responses [37]. Additionally, alterations in tryptophan metabolism, leading to imbalances in serotonin and other neurotransmitter-related metabolite levels, may contribute to IBS symptoms, such as altered bowel habits and mood disturbances [38]. Understanding the intricate interplay between amino acid metabolism and the pathogenesis of IBS is a potential opportunity for developing targeted therapeutic interventions to restore intestinal homeostasis and alleviate symptoms in affected individuals.

Nicotinamide metabolism has been linked to inflammatory bowel-related conditions, specifically the coenzyme nicotinamide adenine dinucleotide (NAD+). NAD+ is a crucial cofactor in various metabolic pathways, including energy metabolism and cellular redox reactions. Nicotinamide riboside, a precursor of NAD+, has garnered attention for its potential therapeutic benefits in intestinal inflammation conditions [39]. Nicotinamide riboside supplementation has been shown to enhance NAD+ levels, improve mitochondrial function, and alleviate inflammation in mouse preclinical models of gastrointestinal disorders [40,41]. Furthermore, NAD+ modulation may influence immune cell function and intestinal microbiota composition, which have been implicated in the pathogenesis of IBS.

For faecal samples, the metabolite differences between the ‘all constipation’ or ‘all diarrhoea’ groups compared to the healthy control group were much less, with weaker differences than plasma. Differentially abundant metabolites in faecal samples included homovanillic acid, which is metabolised from dopamine and linked to neurological disorders, including epilepsy, Parkinson’s disease, and major depression [42], and was higher in healthy controls compared to the ‘all diarrhoea’ group. The role of homovanillic acid is linked to dopamine; however, it has also been found in beer and olives and could, therefore, be a dietary intake by-product.

### 3.3. Strengths and Limitations

A multimodal metabolomic approach utilising the non-polar, semi-polar and polar extracts of plasma and faecal samples is a strength of the research presented. It provides a comprehensive coverage of these metabolites and insights into changes in biochemical processes in the intestinal microbiota and host metabolism of individuals with FGIDs.

However, there are limitations, as the results are limited to metabolites that can be accurately annotated using libraries and databases based on mass and retention time. Annotation was only possible for a few polar and semi-polar metabolites. Averaging across both sample types, only ~11% of polar and semi-polar metabolites and ~20% of lipids could be annotated, which is not unusual in untargeted metabolomics studies [43]. Although some detected features were isotopes and not representative of unique metabolites, the low annotation percentages highlight the complexity of the plasma and faecal metabolome and the origins of the metabolites (dietary, host, and/or microbial sources), making annotation using primarily human databases difficult. Future advances in collecting more MS/MS spectral data and databases that include microbial metabolites will exponentially increase the understanding of host-microbial interactions in FGIDs [44].

The plasma and faecal metabolomes of the ‘all constipation’ and ‘all diarrhoea’ groups were compared separately to healthy controls. Although FGID are an encompassing disorder, it is postulated that the mechanisms of each FGID subtype would differ based on phenotypic aetiologies. The dissimilarity in VIP metabolites was important to separate the ‘all constipation’ or the ‘all diarrhoea’ group from the healthy control group, highlighting the variable and complex symptoms that may reflect different biochemical processes between these groups. Similar to microbiome-based analyses, this heterogeneity makes comparisons and inferences between findings difficult due to the lack of standardisation in analytical methods and statistical approaches [5].

### 3.4. Strategies and Considerations for the Future

FGID involves complex relationships between food intake and symptom triggers. Nutrient composition, including factors such as fibre content and carbohydrate malabsorption, plays a significant role in the onset of symptoms like bloating, abdominal pain, and changes in bowel movements. Diets such as the low FODMAP diet have shown efficacy in reducing symptoms in conditions like irritable bowel syndrome (IBS); the underlying mechanisms of how a low FODMAP diet or other nutritional intervention strategies might work at a molecular level remain under investigation [45,46].

The gut-brain axis is a critical communication network that influences both digestive function and mental health. Disturbances in this axis, including changes in gut microbiota and nutrient absorption, can exacerbate or lead to mental health issues like anxiety, depression, and stress-related disorders. For instance, FGIDs are prevalent in individuals with eating disorders and may perpetuate or arise from disordered eating behaviours [47]. Mechanisms such as nutrient sensing and microbiota changes influence the interaction between the gut and the brain, highlighting the need for further research into how dietary therapies can manage both gastrointestinal and psychiatric symptoms.

Finally, studies like the one described here using untargeted metabolomics, combined with or without data-directed analysis approaches, can generate many metabolic pathways and metabolites that are implied to be involved in the complex disease phenotype described here. Each of these biomarkers should be remeasured from a separate validation cohort, preferably also large in the number of participants, using targeted quantitative analyses to confirm their potential use as biomarkers but also in understanding the opportunities for mitigating the phenotypic disorder through nutrition or drug therapy approaches. This then provides considerable power in not only finding the complex markers within large datasets, but also confirms them by validation with a separate cohort and targeted analytical approaches.

In conclusion, the plasma and faecal metabolomes offer insights into metabolites and pathways that could be important in FGID. Polar and semi-polar analyses found specific metabolites in plasma and faeces with higher or lower relative intensities between the ‘all constipation’ or ‘all diarrhoea’ groups compared to healthy controls. The most significant variation was observed in the plasma metabolome, where differences were observed in the ‘all constipation’, ‘all diarrhoea’ groups or IBS group, compared to healthy controls. MSEA and network analyses highlighted perturbations in amino acid and bile acid metabolism and their impact on purine metabolism. The faecal lipidome also revealed that ceramides and other key lipids differed between groups and could indicate perturbed metabolic processes in the intestinal tract of the participants with constipation or diarrhoea.

## 4. Materials and Methods

### 4.1. Participants

Metabolomic analyses were carried out on samples from the Christchurch IBS cohort to investigate the mechanisms of gut relief and improved transit (COMFORT cohort; universal trial number: U1111-1216-6662 cohort) [48]. Symptomatic cases were individuals with an FGID diagnosed according to the Rome IV criteria (FC, FD, IBS-C, and IBS-D), while healthy controls were asymptomatic individuals. Faecal (*n* = 221) and plasma (*n* = 206) samples from the COMFORT cohort were analysed using a multimodal LC-MS metabolomic approach. This study was approved by the University of Otago Human Ethics Committee (Ref. # H16/094). Further details on participant recruitment are provided in the Supplementary data and the clinical publication of the COMFORT cohort [48].

### 4.2. Standards and Reagents

Internal standards d_4_-alanine, d_2_-tyrosine, d_5_-tryptophan, and d_10_-leucine were purchased from Cambridge Isotope Laboratories, Inc. (Tewksbury, MA, USA) as standards for monitoring polar and semi-polar LC-MS performance. The lipidomics internal standard, 1-palmitoyl-d_31_-2-oleoyl-sn-glycero-3-phosphate (PE 16:0 D_31_/18:1 sodium salt, for monitoring the lipidomics LC-MS performance) was purchased from Avanti^®^ Polar Lipids, Inc. (Birmingham, AL, USA), while ammonium formate and formic acid were purchased from Sigma Aldrich (Auckland, New Zealand). Acetonitrile, methanol, methyl tert-butyl ether, and chloroform of optima LC-MS grade were purchased from Thermo Fisher Scientific (Auckland, New Zealand).

### 4.3. Sample Extraction

*Plasma:* Plasma samples were extracted using biphasic extraction, slightly adapted from a previously reported method [49]. Briefly, 100 μL plasma in a microcentrifuge tube was mixed with 800 μL pre-chilled (−20 °C) CHCl_3_:MeOH (50:50, v/v) containing 10 μg/mL of the internal standards d_4_-alanine, d_2_-tyrosine, d_5_-tryptophan, and d_10_-leucine. The sample was then agitated for 30 s and stored at −20 °C for 60 min to allow protein precipitation, followed by the addition of 400 μL H_2_O, vortex mixed for 30 s, and then centrifuged at 14,000× *g*, 4 °C, for 10 min. Blank samples were prepared following the same protocol, replacing plasma with H_2_O. Two hundred microlitres of the upper aqueous layer was transferred to a tube for polar analysis, a further 200 μL was transferred to another tube for semi-polar analysis, and 200 μL of the lower organic layer was transferred to a tube for lipidomic analysis.

All tubes were evaporated to dryness under a nitrogen stream and stored at −80 °C. To account for intra- and inter-batch variation, pooled quality control (QC) samples were prepared by combining an aliquot of the upper or lower phase from every sample extracted on the same day in a clean glass tube and stored at −80 °C for each of the three metabolomic streams. At the end of all sample extractions, the pooled samples on each day were combined, dispensed into separate 200 μL aliquots and then evaporated to dryness under a nitrogen stream, and stored at −80 °C. On the day of instrumental analysis of the plasma extracts, dried polar, semi-polar, and lipid extracts were reconstituted in 200 μL acetonitrile:H_2_O (50:50, v/v), 200 μL acetonitrile:H_2_O (10:90, v/v), and 200 μL modified Folch solution (CHCl_3_:MeOH:H_2_O, 66:33:1, v/v/v) containing pre-dissolved 0.01% PE(16:0 D_31_/18:1) internal standard [0.01% (%w/v)] for polar, semi-polar, and lipid metabolite analyses, respectively, and transferred to glass HPLC vials containing 250 μL glass inserts.

*Faecal:* Faecal samples were extracted using a biphasic extraction method slightly adapted from a previously reported method [50]. Briefly, samples were freeze-dried under vacuum, ground, and 50 mg weighed and transferred to 2.0 mL microcentrifuge tubes with a ceramic bead for further homogenisation for 1 min using a QIAGEN TissueLyser II (Thermo Fisher Scientific, Auckland, New Zealand). Next, 400 µL of 75% MeOH/MilliQ H_2_O was added, and the tubes were vortexed for 30 s. The samples were sonicated for 2 min and then transferred onto ice for 10 min. Next, 1 mL of MTBE was added, and the samples were agitated on a shaker for 1 h at 4 °C and 450 rpm. MilliQ water (250 µL) was added and the samples were vortexed for 30 s and left to rest for 10 min. Tubes were centrifuged (Eppendorf Centrifuge 5427 R, Eppendorf, Hamburg, Germany) at 14,000× *g* for 25 min at 4 °C, 850 µL of the upper lipid phase was transferred to a new tube to be used for lipid analysis, and 300 µL of MilliQ water was added to the remaining extract and then vortex mixed for 30 s and centrifuged for a further 20 min (14,000× *g*, 4 °C). The remaining polar phase was transferred to a new tube and centrifuged for a further 20 min to remove any fine particles. Then, 300 µL aliquots of the polar phase were transferred into two different tubes for polar and semi-polar metabolite analyses. Finally, the extracts from all three microcentrifuge tubes were evaporated to dryness under nitrogen. On the day of instrumental analysis of the faecal extracts, samples for lipidomic analysis were reconstituted in 500 µL of a 2:1 CHCl_3_:MeOH containing PE(16:0 D_31_/18:1) internal standard at 10 µg/mL concentration and vortexed until all material was redissolved. The samples were centrifuged at 12,000× *g* for 12 min at 4 °C, and 100 µL of the solution was transferred to a glass HPLC vial containing a 250 µL insert. Polar faecal extracts for metabolomic analysis using HILIC were reconstituted in 200 µL of a 50:50 acetonitrile/H_2_O solution and then vortexed until all material dissolved. The extracts were centrifuged at 12,000× *g* for 12 min at 4 °C, and 100 µL of the solution was transferred to a glass HPLC vial containing a 250-glass insert. Faecal sample extracts for semi-polar metabolite (C18) analysis were reconstituted in 200 µL of a 90:10 H_2_O/acetonitrile solution and then vortexed until all material dissolved. The extracts were centrifuged at 12,000× *g* for 12 min at 4 °C, and 100 µL of the solution was transferred to a glass HPLC vial containing a 250 µL insert.

### 4.4. General Mass Spectrometry Analytical Parameters

Polar and semi-polar metabolites were extracted using predominantly aqueous-based solvents and resolved using hydrophilic interaction liquid chromatography (HILIC) and reversed-phase columns, respectively. In contrast, lipids were extracted with an organic solvent and then resolved on a modified reversed-phase column. All metabolomic analyses were conducted on Thermo Fisher LC-MS/MS systems fitted with an Accela 1250 UHPLC pump system (Thermo Fisher Scientific, Waltham, MA, USA) coupled to a PAL autosampler (CTC Analytics AG., Zwingen, Switzerland) and either a Q-Exactive or Exactive MS with electrospray ionisation. All three faecal analyses (polar, semi-polar and lipid) and plasma lipid analyses were conducted on a Q-Exactive to facilitate MS/MS spectral identification. Plasma polar and semi-polar analyses were conducted on an Exactive due to instrument availability; thus. all subsequent data analyses described here were kept separate in their individual sample type (plasma or faecal) and analysis mode (polar, semi-polar or lipid) rather than fully integrated. To ensure mass accuracy of each instrument and analysis mode, positive and negative mass calibrations using Pierce™ LTQ electrospray ionisation (ESI) (Positive and Negative Ion Calibration Solutions, Thermo Fisher Scientific, Waltham, MA, USA) of the Orbitrap systems were completed prior to sample analysis and after every 100 samples by direct infusion. The samples were cooled at 4 °C in an autosampler until sample injection. The samples were split into three analytical batches of approximately 70 samples, with pooled QC and blank extracts injected every 10 samples. Multimodal analyses were conducted as previously described [51,52]. The LC-MS details are provided in the Supplementary data. For annotation purposes, for the plasma lipids and all faecal extracts (all analysed on a Q-Exactive), data-dependent MS/MS was performed on the pooled QC, and 10 randomly selected samples at the end of each batch of samples.

### 4.5. Data Processing and Statistical Analysis

Instrument raw files were converted to the mzML format using MS Convert (ProteoWizard version 3.0.20266) [53]. Peak detection and alignment were performed using XCMS as part of the Bioconductor package for R statistical software (R version 3.6.1) [54] and batch correction was performed using Workflow 4 Metabolomics (version 4.0) [55]. Lipids were annotated using LipidSearch software version 4.1.16, on representative MS^2^ datafiles (Thermo Fisher Scientific, Waltham, MA, USA). In-house libraries were used for the annotation of all polar and semi-polar metabolites. MS Dial (version 4.6) [56] was used to annotate faecal metabolites using tandem MS/MS data collected from polar and semi-polar analytical streams. The human metabolome database [57] was used to identify metabolic features selected from partial least squares—discriminant analysis (PLS-DA) models based on their variable importance in projection (VIP) score.

Principal component analysis (PCA) was first performed on the multivariate data to observe the variance in the datasets. A data-driven analysis approach using PLS-DA was then performed to analyse the different individual LC-MS analysis mode datasets for each sample type. PLS-DA is a discriminating statistical analysis that optimises intergroup differences by rotating PCA components to maximise separation among groups and shed light on variables carrying class-separating information. The PLS-DA models were analysed using SIMCA (version 16.0.1). The quality of the PLS-DA models generated was measured using R2X, R2Y, and Q2, with values closest to one signifying a better-fitting model [58]. Cross-validated ANOVA (CV-ANOVA) was used to test the significance of the PLS-DA models [58]. Models with a positive Q2 value and a CV-ANOVA *p*-value < 0.05 were considered significant and used for further feature selection and model refinement. Multiple models were investigated using a cutoff of approximately 5–10% of the original number of features selected by examining the VIP scores for normality to determine the optimal feature cutoffs for the best (and most significant) models for metabolite and lipid identification. Thus, the number of features and the VIP score cutoff used for the models varied during the data selection step to create the best available model.

In the analytical streams where duplicate measures of the same peak were detected in both positive and negative modes, the negative mode peak was removed, as it is most frequently of a lower intensity. This procedure was performed to reduce the effect of false correlations for the same metabolite.

Metaboanalyst (version 5.0) [59] was used for univariate analysis (*t*-test) and fold-change calculations of the faecal and plasma metabolomes. Multiple testing adjustment was performed on both approaches using the false discovery rate (FDR) method, with an adjusted *p* < 0.05 deemed statistically significant. Metscape (version 3.1) [60] as part of Cytoscape, was used for pathway mapping. VIP plots were formulated to understand the variability and complexity of metabolomic data, mitigate the false discovery of an overfitting model, and investigate metabolites that contribute to data separation for PLS-DA analysis.

Samples were grouped into major functional types, i.e., all diarrhoea or all constipation, to enable examination at the broader disorder level first, and then subsequently we analysed the deeper levels of subgroups as well e.g., FC and IBS-C separately. This allows for a comprehensive and thorough analysis of broad and specific functional disorders within the metabolomic dataset.

## Figures and Tables

**Table 1:** Plasma lipids with significantly different relative intensities (*p* < 0.05) and a fold-change > ±1.3 between ‘all constipation’ (FC + IBS-C) and healthy controls (HC).

Lipid Species	Fold Change	log2(FC)	*p*-Value
PE (16:0/22:6)	0.722	−0.469	0.006
TG (18:1/18:1/18:2)	1.301	0.380	0.044
TG (61:3)	1.329	0.411	0.030
TG (59:2)	1.382	0.467	0.004
TG (60:6)	1.533	0.616	0.045

A negative log fold change (log2FC) value indicates higher relative intensities in the ‘all constipation’ group compared to the healthy control. Conversely, a positive log2FC value indicates lower relative intensities in the ‘all constipation’ group compared to the healthy group. Lipid abbreviations are defined in Supplementary Table S1. Common lipid nomenclature: Lipid species followed by total carbon number:number of double bonds.

**Table 2:** Plasma lipids with significantly different relative intensities (*p* < 0.05) and a fold-change > ±1.3 between IBS-constipation (IBS-C) and healthy controls (HC).

Lipid Species	Fold Change	log2(FC)	*p*-Value
PE (16:0/22:6)	0.600	−0.737	<0.001
PC (36:6)	0.678	−0.560	0.002
PC (34:4)	0.681	−0.555	0.002
SM (d22:0/18:2)	0.683	−0.549	<0.001
PC (38:7)	0.690	−0.535	<0.001
PI (34:3)	0.695	−0.525	<0.001
PC (32:1)	0.695	−0.524	0.007
PC (32:2)	0.715	−0.484	0.007
PE (160/20:4)	0.725	−0.463	0.007
PS (39:5)	0.729	−0.457	0.007
PE (16:0/20:4)	0.737	−0.441	0.003
PS (37:3)	0.738	−0.438	0.006
PC (37:6)	0.751	−0.414	0.016
PC (40:7)	0.753	−0.409	0.014
PG (36:2)	0.759	−0.398	0.004
PC (16:0/16:1)	0.759	−0.397	0.022
PI (37:1)	0.761	−0.395	0.002
SM (d41:2)	0.763	−0.389	0.007
PS (43:6)	0.767	−0.383	0.003
TG (16:0/18:1/18:1)	1.313	0.393	0.038
TG (16:1/17:0/18:1)	1.339	0.421	0.035
TG (16:0/18:1/18:2)	1.356	0.440	0.047
TG (58:1)	1.371	0.455	0.048
TG (17:0/18:1/18:1)	1.457	0.543	0.019
TG (59:2)	1.466	0.552	0.020

A negative log fold change (log2FC) value indicates higher relative intensities in the IBS-constipation group compared to the healthy control group. Conversely, a positive log2FC value indicates lower relative intensities in the IBS-constipation group compared to the control group. Lipid abbreviations are defined in Supplementary Table S1. Common lipid nomenclature: Lipid species followed by total carbon number:number of double bonds.

**Table 3:** Twenty-four faecal lipids with significantly different relative intensities (*p* < 0.05) and a fold-change > ±1.3 between the ‘all constipation’ (FC + IBS-C) group and healthy controls (HC).

Lipid Species	Fold Change	log2(FC)	*p*-Value
Cer (d18:0/12:0)	0.323	−1.628	0.015
Cer (d18:1/12:0)	0.438	−1.192	0.027
TG (6:0/15:0/20:5)	0.592	−0.757	0.024
LPG (31:1)	0.608	−0.718	0.045
PE (34:0)	0.658	−0.604	0.032
TG (41:6)	0.661	−0.597	0.034
Cer (d17:0/15:0)	0.682	−0.552	0.042
Cer (d16:0/16:0+O)	0.689	−0.537	0.032
Cer (d17:0/16:0)	0.711	−0.491	0.040
Cer (d15:0/16:0+O)	0.719	−0.475	0.011
Cer (d16:1/16:0+O)	0.734	−0.445	0.037
Cer (d15:0/16:0+O)	0.736	−0.442	0.021
Cer (d18:0/17:0+O)	0.754	−0.406	0.031
SM (d36:4)	1.727	0.788	0.046
DG (18:1/18:2)	1.763	0.818	0.037
TG (12:0 p/8:0/16:2)	1.767	0.821	0.037
DG (16:0/18:1)	1.886	0.916	0.021
TG (18:1/18:1/22:0)	1.933	0.951	0.032
DG (18:1/18:1)	1.974	0.981	0.026
Pet (19:1/18:1)	2.276	1.187	0.011
DG (18:0/18:1)	2.302	1.203	0.027
DG (22:0/18:2)	2.521	1.334	0.034
DG (18:1/22:0)	4.334	2.116	0.032
TG (4:0/18:2/18:2)	7.361	2.880	0.015

A negative log fold change (log2FC) value indicates higher relative intensities in the ‘all constipation’ (FC + IBS-C) group compared to the healthy control. Conversely, a positive log2FC value indicates lower relative intensities in the constipation group compared to the control group. Lipid abbreviations are defined in Supplementary Table S2. Common lipid nomenclature: Lipid species followed by total carbon number:number of double.

**Table 4:** Faecal semi-polar metabolites with significantly different relative intensities (*p* < 0.05) and a fold-change > ±1.3 between ‘all constipation’ (FC + IBS-C) and healthy controls (HC).

Semi-Polar Metabolite	Fold Change	log2(FC)	*p*-Value
Glycocholic acid	0.489	−1.032	0.029
Everninic acid	0.557	−0.845	0.011
Spermidine	0.662	−0.596	0.004
Nicotinic acid	0.731	−0.453	0.002
Choline	0.739	−0.437	0.045
Glycine-Tyrosine	0.740	−0.435	0.019
Pimelic acid	1.435	0.521	0.018

A negative log fold change (log2FC) value indicates higher relative intensities in the ‘all constipation’ (FC + IBS-C) compared to the healthy control group. Conversely, a positive log2FC value indicates lower relative intensities in the ‘all constipation’ group compared to the healthy control group.

**Table 5:** Faecal polar metabolites with significantly different relative intensities (*p* < 0.05) and a fold-change > ±1.3 between ‘all constipation’ (FC + IBS-C) and healthy controls (HC).

Polar Metabolite	Fold Change	log2(FC)	*p*-Value
N-Acetyl-D-galactosamine	0.690	−0.536	0.005
Glutamic acid	0.743	−0.428	0.016
N-alpha-acetyl-l-lysine	0.734	−0.435	0.035
Ala-leu	0.766	−0.385	0.043

A negative log fold change (log2FC) value indicates higher relative intensities in the ‘all constipation’ (FC + IBS-C) compared to the healthy control. Conversely, a positive log2FC value indicates lower relative intensities in the constipation group compared to the healthy control group.

**Table 6:** Faecal lipids with significantly different relative intensities (*p* < 0.05) and a fold-change > ±1.3 between the ‘all diarrhoea’ (FD + IBS-D) group and healthy controls (HC).

Lipid Species	Fold Change	log2(FC)	*p*-Value
Cer (d18:1/12:0)	0.432	−1.211	0.028
TG (18:0/16:0/16:0)	0.435	−1.200	0.002
MGDG (16:0/18:3)	0.442	−1.177	0.046
TG (16:0/16:0/16:0)	0.445	−1.167	0.019
Cer (d18:0/12:0)	0.446	−1.164	0.014
Cer (d18:1/18:0+O)	0.463	−1.111	0.012
ChE (18:1)	0.526	−0.926	0.006
TG (18:0/16:0/18:0)	0.560	−0.838	0.017
Cer (d18:1/18:0)	0.597	−0.745	0.033
PE (15:0/14:0)	0.617	−0.698	0.027
DG (16:0/16:0)	0.657	−0.605	0.013
DG (18:0/16:0)	0.661	−0.598	0.002
Cer (d17:1/24:0)	0.681	−0.555	0.040
Cer (d18:0/24:0)	0.698	−0.519	0.044
Cer (d18:1/18:2)	0.722	−0.470	0.033
Cer (d18:0/22:0)	0.732	−0.450	0.049
Cer (d42:1+O)	0.732	−0.450	0.034
Cer (d18:1/16:0)	0.754	−0.407	0.047
OAHFA (41:2)	1.851	0.888	0.022

A negative log fold change (log2FC) value indicates higher relative intensities in the ‘all diarrhoea’ (FD + IBS-D) group compared to the healthy control group. Conversely, a positive log2FC value indicates lower relative intensities in the ‘all diarrhoea’ group compared to the control group. Lipid abbreviations are defined in Supplementary Table S2. Common lipid nomenclature: Lipid species followed by total carbon number:number of double bonds.

**Table 7:** Significant pathways and impact values from comparing plasma polar and semi-polar metabolites using metabolite set enrichment analysis for healthy controls vs. IBS-D.

Pathway	*p*-Value	Impact
Aminoacyl-tRNA biosynthesis	1.18 × 10^−11^	0.113
Nitrogen metabolism	9.91 × 10^−7^	0.008
Alanine, aspartate and glutamate metabolism	1.95 × 10^−5^	0.412
Phenylalanine, tyrosine and tryptophan biosynthesis	5.79 × 10^−4^	0.008
Glycine, serine and threonine metabolism	6.05 × 10^−4^	0.233
Arginine and proline metabolism	7.98 × 10^−4^	0.313
D-arginine and D-ornithine metabolism	0.006	0
Valine, leucine and isoleucine biosynthesis	0.007	0.047
beta-Alanine metabolism	0.008	0.257
Cysteine and methionine metabolism	0.009	0.050
D-glutamine and D-glutamate metabolism	0.011	0.027
Pyrimidine metabolism	0.011	0.036
Propanoate metabolism	0.014	0.114
Cyanoamino acid metabolism	0.023	0
Phenylalanine metabolism	0.028	0.168
Taurine and hypotaurine metabolism	0.035	0.363
Thiamine metabolism	0.049	0.125

**Table 8:** Faecal semi-polar metabolites with significantly different relative intensities (*p* < 0.05) and a fold-change > ±1.3 between the ‘all diarrhoea’ (FD + IBS-D) group and healthy controls (HC).

Semi-Polar Metabolite	Fold Change	log2(FC)	*p*-Value
Everninic acid	0.654	−0.613	0.050
Uric Acid	0.658	−0.605	0.029
Azelaic acid	0.742	−0.430	0.040
Thymidine	1.300	0.379	0.049
Adenosine	1.360	0.444	0.026
Quinoic acid	2.023	1.017	0.019
2-Piperidone	2.228	1.156	0.006
Homovanillic acid	3.549	1.827	0.026

A negative log fold change (log2FC) value indicates higher relative intensities in the ‘all diarrhoea’ (FD + IBS-D) compared to the healthy control group. Conversely, a positive log2FC value indicates lower relative intensities in the diarrhoea group compared to the control group.

**Table 9:** Faecal semi-polar metabolites with significantly different relative intensities (*p* < 0.05) and a fold-change > ±1.3 between the ‘all diarrhoea’ (FD + IBS-D) group and healthy controls (HC).

Polar Metabolite	Fold Change	log2(FC)	*p*-Value
n-Acetylputrescine	0.378	−1.405	0.020
4-methyl-5-thiazoleethanol	0.454	−1.140	0.012
Guanosine	0.570	−0.812	0.002
2-Piperidone	0.586	−0.772	0.045
Cytarabine	0.666	−0.585	0.011
Benzaldehyde	0.688	−0.539	0.025
Indoline	0.729	−0.456	0.018
Guanine	0.732	−0.449	0.030
3-Methylpyrazole	0.743	−0.429	0.013
Adenosine	0.753	−0.409	0.033

A negative log fold change (log2FC) value indicates higher relative intensities in the ‘all diarrhoea’ (FD + IBS-D) compared to the healthy control group. Conversely, a positive log2FC value indicates lower relative intensities in the diarrhoea group compared to the control group.

## Data Availability

Data will be fully available by request to the corresponding authors.

## Supplementary Materials

The following supporting information can be downloaded at: https://www.mdpi.com/article/10.3390/ijms252413465/s1.
