# Supplementary material for: Characterisation of the Plasma and Faecal Metabolomes in Participants with Functional Gastrointestinal Disorders
Source: Int J Mol Sci. 2024 Dec 16;25(24):13465. doi: 10.3390/ijms252413465 (PMC11677738; doi:10.3390/ijms252413465)

Supplementary Data – COMFORT metabolomics manuscript.

Methods:

Participants:

Symptomatic participants undergoing colonoscopy for symptom investigation or surveillance aged 18–70 years were recruited in Christchurch, New Zealand. Healthy controls were asymptomatic individuals undergoing colonoscopy for surveillance due to a family history of colorectal cancer, personal history, or screening for colorectal cancer or polyps aged 18–70 years. Pregnant women or individuals with a known organic disorder (inflammatory bowel disease, colorectal cancer, or diverticulitis), previous bowel resection, and coeliac disease were excluded from the study.

Lipidomic liquid-chromatography mass-spectrometry analysis

Five µL of plasma or faecal extract were injected into a 2 µL injection loop maintained at 65 °C with a flow rate of 600 µL/min and separated on an Acquity CSH™ C18 column 1.7 µm, 2.1 mm × 100 mm (Waters, Milford, MA, USA). Solvent A was 60% acetonitrile in water with 10 mM ammonium formate and 0.1% formic acid. Solvent B was 90% iso-propanol in acetonitrile with 10 mM ammonium formate and 0.1% formic acid. Gradient elution started at 15% B, increasing to 30% B at 2 min, 48% B at 2.50 min, 82% B at 11 min, then 99% B at 11.50, maintained until 14.10 min, reduced to 15% B and held there for 3 min for equilibration before next sample injection. Mass spectral detection at 35,000 resolution was performed in positive and negative ionisation modes with ESI over 15 min and a mass range from 200 to 2000 *m/z*. Data-dependant MS/MS was performed on the QC and 10 randomly selected samples at the end of the batch to assist with lipid annotation.

Polar metabolomic liquid-chromatography mass-spectrometry analysis

Five µL of plasma or faecal extract was injected into a 2 µL injection loop and eluted on SeQuant® ZIC®-pHILIC column (100 x 2.1mm x 5µm, PEEK coated, Merck KGaA, Darmstadt, Germany), attached to a SeQuant® ZIC®-pHILIC Guard (20 x 2.1mm, PEEK coated, Merck KGaA, Darmstadt, Germany), and a KrudKatcher™ ULTRA HPLC In-Line Filter (0.004in x 0.5µm, Torrance, CA, USA). A 250 µL/min flow rate was maintained with solvent A 0.1% formic acid in acetonitrile and solvent B 16 mM ammonium formate in water. Gradient elution started at 3% B, increasing to 30% B at 12 min, 90% B at 14.50 min and held there till 18.50 min, where it returned to 3% B until 24 min for equilibration before the next sample injection. Mass spectral detection at 35,000 resolution for faecal extracts and 25,000 for plasma extracts was performed in positive and negative ionisation modes with ESI over 19 min, with a mass range from 55 to 825 *m/z*. For the faecal samples (analysed on a Q-Exactive), data-dependant

MS/MS was performed on the QC, and 10 randomly selected samples at the end of the batch to assist with annotation.

Semi-polar metabolomic liquid-chromatography mass-spectrometry analysis

Five µL of plasma or faecal extract were injected into a 2 µL injection loop and eluted on a Hypersil GOLD column (2.1 mm x 100 mm x 1.9 µm, Thermo Fisher Scientific, Waltham, MA, USA) with a 400 µL/min flow rate. Solvent A was 0.1% formic acid in the water, and solvent B was 0.1% formic acid in acetonitrile. Gradient elution started at 0% B, increasing to 100% B at 11 min, held for 3 min and then back to 0% B at 14 min and held for 2 min for equilibration before the next sample injection. Mass spectral detection at 35,000 resolution for faecal and 25,000 resolution for plasma was performed in positive and negative ionisation modes with ESI over 14 min, with a mass range from 80 to 1200 *m/z*.

Table S1: Lipid groups annotated from plasma sample analysis with corresponding common abbreviation and number of lipids detected from each group.

Lipid Group	Lipid Abbreviation	Lipids in Group
Ceramide	Cer	9
Cholesterol ester	Che	8
Diglyceride	DG	22
Dimethylphosphatidylethanolamine	dMePE	1
Lysophosphatidylcholine	LPC	3
Phosphatidylcholine	PC	102
Phosphatidylethanolamine	PE	30
Phosphatidylglycerol	PG	6
Phosphatidylinositol	PI	23
plasmenyl-phosphatidylcholine	pPC	3
plasmenyl-phosphatidylethanolamine	pPE	11
Phosphatidylserine	PS	32
Sphingomyelin	SM	61
Sterol	ST	8
Triglyceride	TG	104
Zymosteryl	ZyE	5

Table S2: Lipid groups annotated from faecal sample analysis with corresponding common abbreviation and number of lipids detected from each group.

Lipid Group	Lipid Abbreviation	Lipids in Group
Ceramide	Cer	74
Cholesterol ester	Che	4
Diglyceride	DG	102
Digalactosyldiacylglycerol	DGDG	7
Lysophosphatidylcholine	LPC	3
Lysophosphatidylethanolamine	LPE	3
Lysophosphatidylglycerol	LPG	1
Monoglyceride	MG	9
Monogalactosyldiacylglycerol	MGDG	11
(*O*-Acyl)-ω-hydroxy fatty acids	OAHFA	17
Phosphatidylcholine	PC	8
Phosphatidylethanolamine	PE	13
Phospahtidylethanol	PEt	5
Phosphatidylglycerol	PG	17
Phosphatidylinositol	PI	4
Phosphatidylserine	PS	3
Sitosteryl ester	SiE	1
Sphingomyelin	SM	6
Sphingoshine	So	1
Sulfoquinovosyl diacylglycerol	SQDG	1
Stigmasteryl ester	StE	1
Triglyceride	TG	128
Zymosteryl	ZyE	2

Table S3: Semipolar metabolites detected in plasma samples.

S-Methylcysteine sulfoxide	L-Phenylalanine	Citric acid
1,3-Octadiene	L-Proline	Citrulline
2,6-Dimethoxy-4-methylphenol	L-Serine	Creatine
2-[(Methylthio)methyl]-2-utenal	L-Threonine	D-Tryptophan
3,4-Dihydroxybenzylamine	L-Tryptophan	Glutaric acid
3-Amino-2-piperidone	L-Tyrosine	Glyceric acid
3-Indoleacetonitrile	Methylisopelletierine	Glycocholic acid
3-Methoxybenzenepropanoic acid	Myristoleic acid	Hexanoylglycine
4,5-Dihydro-2-methylthiazole	N-(3-Methylbutyl)acetamide	Hippuric acid
4-Hydroxycitrulline	N,N-Dimethylaniline	Hypoxanthine
4-Hydroxynonenal	N-Acetylarylamine	Indolelactic acid
4-Hydroxyproline	N-Acetyl-L-leucine	Indoxyl sulfate
5-Phenylvaleric acid	N-Decanoylglycine	Inosine
5Z-Dodecenoic acid	N-Methylphenylethanolamine	L-Glutamic acid
Allantoic acid	N-Nonanoylglycine	L-Glutamine
Azelaic acid	N-Undecanoylglycine	L-Isoleucine
beta-Alanine	Ocimene	L-Malic acid
Betaine	Oleic acid	L-phenylalanine
Capryloylglycine	Ornithine	L-Tyrosine
Carbimazole	Oxoglutaric acid	Methylmalonic acid
Chenodeoxycholate	Palmitoleoyl Ethanolamide	N-Heptanoylglycine
cis-Aconitic acid	Phenylacetic acid	N-Nonanoylglycine
Citric acid	Phenylpyruvic acid	o-OH Phenyl Acetic acid
Citrulline	Pipecolic acid	Oxoglutaric acid
Creatinine	Pyridoxamine	Paracetamol sulfate
Deoxyuridine	Pyruvatoxime	p-Cresol
Dimethylglycine	Sebacic acid	p-Cresol sulfate
Glycyl-Serine	Taurine	Phenol sulphate
Hexadecanedioic acid	Tyramine	Phosphocreatinine
Hydrocinnamic acid	Ubiquinone-1	Phosphonic acid
Hydrogen phosphate	Uracil	Pyroglutamic acid
Hydroxykynurenine	Ureidosuccinic acid	Pyruvaldehyde
Indoleacrylic acid	Valerenic acid	Pyruvic acid
Kynurenic acid	2,3-Dimethylmaleate	Salicylic acid
L-3-Aminodihydro-2(3H)-furanone	2-Ketobutyric acid	Sebacic acid
L-Arginine	2-Octenedioic acid	Suberic acid
L-Carnitine	3-Furoic acid	Taurine
L-Glutamine	4-Hydroxyproline	Thiamine
L-Histidine	Acetylglycine	Uridine
L-Kynurenine	Ascorbic acid	Veratric acid
L-Lysine	Azelaic acid	Xanthine
L-Methionine	beta-Alanine	

Table S4: Semipolar metabolites detected in faecal samples.

(2-oxo-2,3-dihydro-1H-indol-3-yl)acetic acid	Deoxyguanosine	N-acetyl-L-tyrosine
(2R)-6-methylpiperidine-2-carboxylic acid.1	Dioctyl Phthalate	n-acetylneuraminic acid
(E)-3-Methylglutaconic acid	D-Malic Acid	nicotinic acid
1,3,7 Trimethlyuric acid	D-leucine.1	N-omega-Acetylhistamine
2 6-OH Benzoic acid	D-Norleucine	o-Hydroxyhippuric acid-H-
2,3-dihydro-1H-carbazol-4(9H)-one	Dihydroxybenzaldehyde	omega-hydroxydodecanoate
2-Hydroxyisocaproic Acid	Everninic acid	Pantothenic acid
2-hydroxyphenylacetic acid	Feruloylquinic acid (fragment2)	Paracetamol
2-Isopropylmalic acid	Gabapentin	p-coumaric acid
2-methylpyrrolidine	Galacitol	Phenylacetaldehyde.1
2-Piperidone.1	Gluconate	Phthalic acid
3 4 5-triOME benzoic acid	Glucuronic Acid	Phthalic Anhydride
3-(2,4-Dihydroxyphenyl)propionic acid	Glutamic Aci.1	pimelic acid
3-(4-Hydroxyphenyl)propionic acid	Glutaric Acid.1	Pro-Leu_-H-
3,4-Dimethoxybenzaldehyde	Glycl-L-Proline	Proline
3-Hydroxymethylglutarate	Glycocholic acid	Propenyl thiosulfate
3-Methylglutaric acid	Glycoursodeoxycholic acid	Pyroglutamic Acid
4-hydroxybenzaldehyde	Glycraldehyde	Quinoic acid
5-Aminonaphthalene-2-sulfonic acid	Gly-Tyr	Riboflavin
5-aminosalicylic acid	Gly-Val	Ribose
5-Aminovaleric acid betaine	Hippurate	rosmarinate
5-Hydroxyindole-3-acetic-acid	Homovanillic acid	Saccharin
5-Methoxypsoralen	Hypoxanthine.1	Sebacic acid
6-Hydroxynicotinic Acid	Indole-3-carboxcylic acid	Serylleucine
Acetylproline	Indole-3-carboxyaldehyde	Spermidine
Adenosine.1	Inosine.1	Suberic acid
Adipic acid	Isovaleroylglycine	Succinic Acid
Agmatine Sulfate	Ketoleucine	Sugar
Alanylphenylalanine	Kynurenic Acid.1	Taurine
Aminobenzoic acid	L-5-Oxoproline	tauro-deoxycholic acid
Aminosalicylic acid 4	L-Arginine	TCA sodium salt
Aniline	L-Carnitine.1	Theophylline.1
Arabinose	L-Glutamic Acid	Threonine.1
Arginine	LL-2.6-DIAMINOHEPTANEDIOATE_-H-CO2	Thymidine
Aspartic Acid	L-Methionine	Thymine
Azelaic acid	l-phenylanine	Tricarballylic Acid
Azelate	L-tryptophan	Trptophan

Caffeine	L-Tyrosine.1	Tyramine.1
Carbamazepine	Metformin	Tyrosine
Cholic acid	Methioninesulfoxide	undecanedioic_acid_-H-
Choline	Methyl-2-hydroxyisobutyric acid	Uracil
Citrulline	Metoprolol	Uric Acid
Coumaroylquinic	N,N-Dimethylglycine.1	uridine
Coumaroylquinic acid2(fragment2)	N-acetyl Mesalazine	Urocanate
Creatinine.1	n-acetylleucine	Xanthine.1
D-Ala-D-Ala.1	N-Acetyl-L-methionine	XANTHURENATE_-H-CO2
Delta-Undecalactone	N-Acetyl-L-phenylalanine	

Table S5: Polar metabolites detected in plasma samples.

L-Glutamic acid	Pyruvaldehyde	2-Furancarboxaldehyde
5-Aminopentanamide	Putrescine	Sulfate
L-Methionine	Urea	Orthophosphate
2-Piperidinone	2-Oxo-4-methylthiobutanoic acid	L-Serine
L-Histidine	Gamma-amino-n-butyric acid	Glyceric acid
L-Isoleucine	Trimethylamine N-oxide	Methylphosphate
N(6)-Methyllysine	N-Methylnicotinamide	Acetylglycine
L-Asparagine	Proline betaine	Succinate
Creatine	Trans-4-hydroxy-L-proline	L-Homoserine
5,6-Dihydrouridine	2-Ketobutyric acid	3,4-Dihydroxybutyric acid
Taurine	Glycerophosphocholine	Taurine
L-Serine	Pipecolic acid	Pyroglutamic acid
L-Lysine	L-Leucine	Glutaconic acid
L-Proline	N-Acetylputrescine	Glutaconic acid
Glycine	Trigonelline	L-Asparagine
L-Tryptophan	Creatinine	L-Aspartic acid
Indoleacrylic acid	Piperidine	Hypoxanthine
L-Alanine	Indoleamine	L-Glutamine
Hypoxanthine	Cytosine	L-Glutamic acid
Dihydrothymine	L-Threonine	Citramalic acid
L-Tyrosine	Methyl propenyl ketone	Citramalic acid
L-Valine	DL-2-Aminooctanoic acid	L-Histidine
Isonipecotic acid	Ethanolamine	Arabinonic acid
Pyroglutamic acid	Diethanolamine	Quinolinic acid
L-phenylalanine	3-Dehydroxycarnitine	Uric acid
Ornithine	O-methyl threonine	Shikimic acid
L-Arginine	Thiocyanate	Suberic acid

L-Carnitine	Glyoxylic acid	L-Arginine
L-Glutamine	Propionic acid	L-Citrulline
Dimethylglycine	Phosphonic acid	Diphosphate
Citrulline	beta-Alanine	D-Glucose
Guanidine		

Table S6: Polar metabolites detected in faecal samples.

(2R)-6-methylpiperidine-2-carboxylic acid	d-norvaline	Morpholine
2,3 Dihydro-1H-carbazol-4(9H)-one	D-Pyroglutamic acid	N,N-Dimethylglycine
2-deoxyinosine	d-tryptohpan	N-Acetyl-D-galactosamine
2-Piperidone	Gabapentin	N-acetylglutamic acid
2-Pyrrolidone-5-carboxylic acid	Glutamic acid	n-Acetylputrescine
3-Aminoacetophenone	Glutamine	Nalpha-acetyl-l-lysine
3-hydroxy-1,2-dimethylpyridin-4(1H)-one	glutaric acid	O-Methyl threonine
3-Methylpyrazole	Guanine	p-aminohippuric acid
4-Aminophenol	guanosine	Phenylacetaldehyde
4-methyl-5-thiazoleethanol	gulonic acid gamma-lactone	proline
5-oxo-l-proline	Heptanethiol	propachlor
Adenine	Hypoxanthine	Pyridoxine
Adenosine	Indoline	R-(-)-phenylephrine
Ala-leu	Inosine	Serine
alanine beta	Isonipecotic Acid	shikimic acid
Allysine	Kynurenic acid	Theophylline
Atenolol	L-carnitine	Threonine
Benzaldehyde	L-isoleucine	Trans-4-hydroxy-L-proline
bis(2-hydroxyproyl)amine	L-phenylalnine	Triethanolamine
Creatinine	L-tert-leucine	Trigonelline
Cytarabine	l-theanine	Tyramine
cytosine	L-threanine	tyrosyl-leucine
D-Ala-D-ala	L-tyrosine	Urocanic acid
Deoxycytidine	maleic acid	Valeramide
D-isoleucine	Methionine	Valine
D-leucine	Minoxidil	Xanthine

Table S7: Polar and semi-polar annotated plasma metabolites used for metabolite set enrichment analysis for IBS-C vs healthy control comparison.

Malic acid	4-Hydroxy-L-glutamic acid	Uric acid
Niacinamide	Succinic acid	Imidazolone
Veratric acid	Quinolinic acid	Choline
2-[(Methylthio)methyl]-2-butenal	Citric acid	Acetoin
Carbimazole	Adipic acid	5-Aminolevulinic acid
Indoleacrylic acid	Glycerol 3-phosphate	N-Acetylasparagine
L-Kynurenine	1,3,7-Trimethyluric acid	Propionic acid
L-Phenylalanine	Glycine	Creatinine
L-Tryptophan	L-Lactic acid	Cytosine
1-Deoxy-D-glucitol	beta-Alanine	Dihydrothymine
N6-Acetyl-L-lysine	Citramalic acid	Guanidine
Uridine	Homoserine	Proline
Palmitic acid	Thiocyanate	Putrescine

Supplementary Figure S1: Pathway mapping for plasma polar and semipolar metabolites differentiating the healthy control group from the IBS-C group.

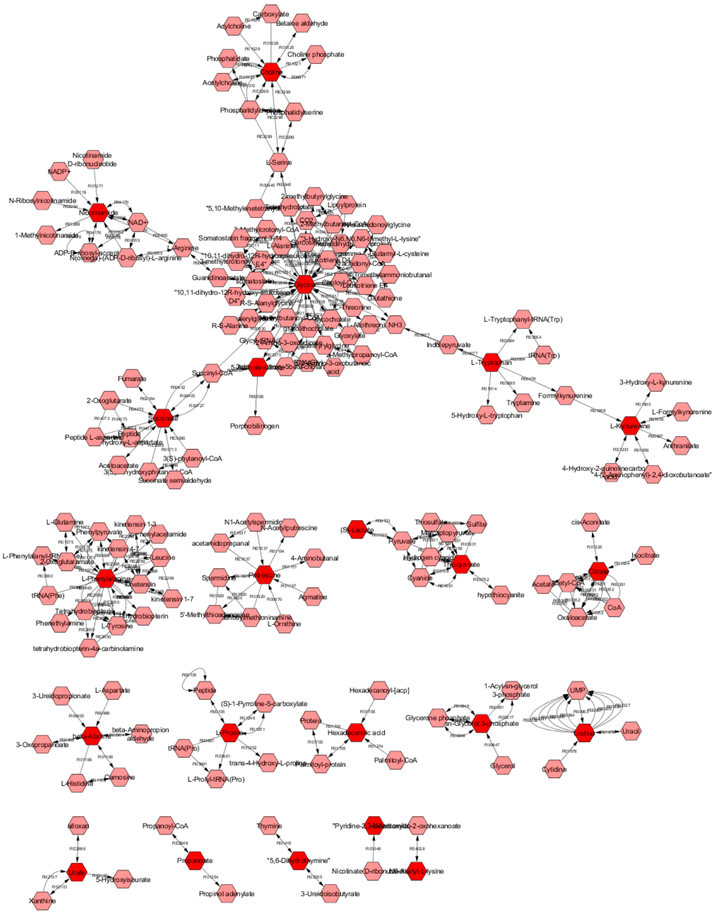

Table S8: Polar and semi-polar annotated plasma metabolites used for metabolite set enrichment analysis for IBS-D vs healthy control comparison.

p-Cresol	Phenylpyruvic acid	2-Ketobutyric acid
Thiamine	Pyridoxamine	3-Dehydroxycarnitine
Indoleacrylic acid	Malic acid	Creatine
L-Carnitine	3-Methyluric acid	Creatinine
L-Phenylalanine	L-Alanine	Cytosine
L-Threonine	beta-Alanine	Gamma-amino-n-butyric acid
L-Tryptophan	Citramalic acid	L-Asparagine
L-Tyrosine	3-Amino-2-piperidone	L-Glutamine
L-Histidine	Methylacetoacetic acid	L-Lysine
N6-Acetyl-L-lysine	Taurine	L-Methionine
L-Arginine	Dihydrothymine	L-Serine
alpha-CEHC	Mevalonic acid	L-Valine
N-Acetylarylamine	N-Acetylglutamine	Ornithine
Oxoglutaric acid	Ibuprofen	Pyroglutamic acid

Supplementary Figure S2: Pathway mapping for plasma polar and semipolar metabolites differentiating the healthy control group from the IBS-D group.

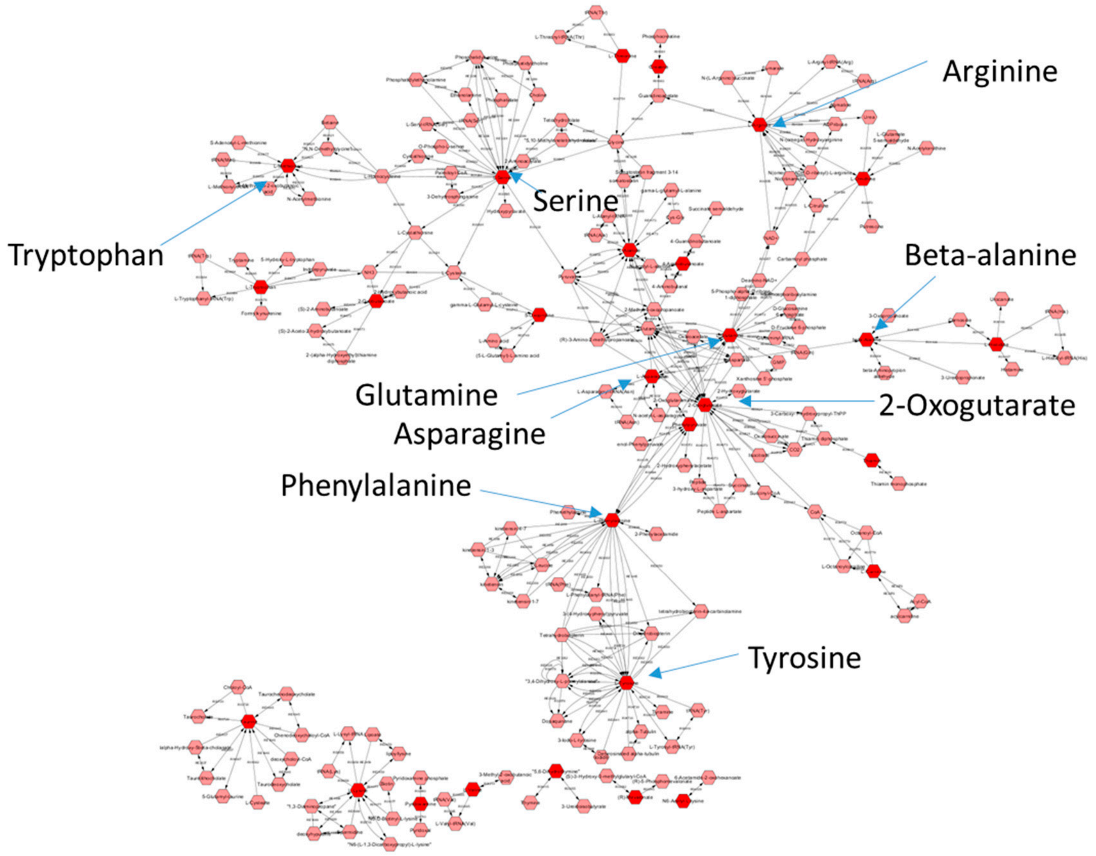

Supplement: Supplementary file 1 [file ijms-25-13465-s001.zip › ijms-3329673-supplementary.pdf]
